# Vascularization in Bioartificial Parenchymal Tissue: Bioink and Bioprinting Strategies

**DOI:** 10.3390/ijms23158589

**Published:** 2022-08-02

**Authors:** Gabriel Alexander Salg, Andreas Blaeser, Jamina Sofie Gerhardus, Thilo Hackert, Hannes Goetz Kenngott

**Affiliations:** 1 Department of General-, Visceral-, and Transplantation Surgery, University Hospital Heidelberg, D-69120 Heidelberg, Germany; thilo.hackert@med.uni-heidelberg.de; 2 Institute for BioMedical Printing Technology, Technical University Darmstadt, D-64289 Darmstadt, Germany; blaeser@idd.tu-darmstadt.de (A.B.); gerhardus@idd.tudarmstadt.de (J.S.G.); 3 Center for Synthetic Biology, Technical University Darmstadt, D-64289 Darmstadt, Germany

**Keywords:** tissue engineering, regenerative medicine, bioprinting, vascularization, biomaterial, bioink, additive manufacturing, bioartificial organs

## Abstract

Among advanced therapy medicinal products, tissue-engineered products have the potential to address the current critical shortage of donor organs and provide future alternative options in organ replacement therapy. The clinically available tissue-engineered products comprise bradytrophic tissue such as skin, cornea, and cartilage. A sufficient macro- and microvascular network to support the viability and function of effector cells has been identified as one of the main challenges in developing bioartificial parenchymal tissue. Three-dimensional bioprinting is an emerging technology that might overcome this challenge by precise spatial bioink deposition for the generation of a predefined architecture. Bioinks are printing substrates that may contain cells, matrix compounds, and signaling molecules within support materials such as hydrogels. Bioinks can provide cues to promote vascularization, including proangiogenic signaling molecules and cocultured cells. Both of these strategies are reported to enhance vascularization. We review pre-, intra-, and postprinting strategies such as bioink composition, bioprinting platforms, and material deposition strategies for building vascularized tissue. In addition, bioconvergence approaches such as computer simulation and artificial intelligence can support current experimental designs. Imaging-derived vascular trees can serve as blueprints. While acknowledging that a lack of structured evidence inhibits further meta-analysis, this review discusses an end-to-end process for the fabrication of vascularized, parenchymal tissue.

## 1. Introduction

**Figure 1:**
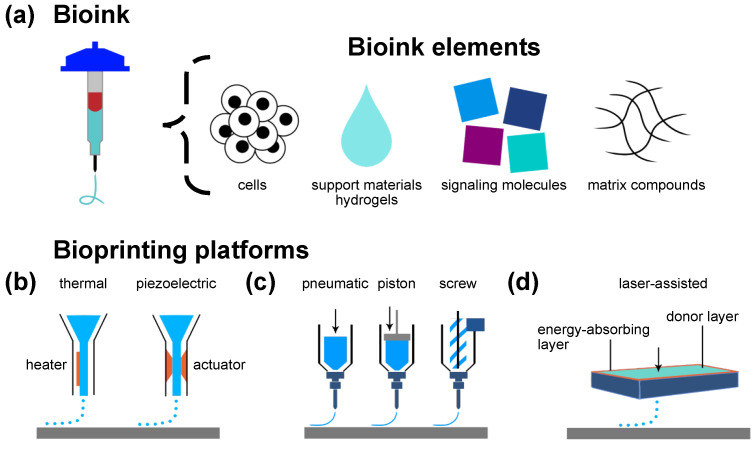
Overview of bioink elements and common bioprinting platforms. (**a**) Bioink composition. The main elements of bioinks are cells, support materials—mostly hydrogels—signaling molecules, and matrix compounds. (**b**) Jet-based drop-on-demand bioprinting enables droplet-based bioink deposition. Droplet formation and deposition can be induced, for example, thermally induced air pressure or piezoelectric pressure. (**c**) Microextrusion bioprinting is based on bioink filament extrusion to fabricate constructs in a layer-by-layer fashion. Bioink extrusion is facilitated by pneumatic or mechanical forces (piston or screw system). (**d**) Laser-assisted bioprinting enables bioink deposition on a receiving substrate layer by pulsed-laser-induced forward transfer (arrow) via an energy-absorbing layer.

Fabrication biotechnology and tissue-engineering research have opened a future perspective for bioartificial organ fabrication. A critical global shortage of donor organs requires the development of alternative treatment strategies [1]. Even though current research provides opportunities to extend the donation pool to post mortem donation after unexpected circulatory death by extracorporeal membrane oxygenation (ECMO), there is still a supply and demand mismatch [2,3]. Tissue engineering envisions potentially replacing or rather supplementing the current gold-standard allograft tissue transplantation and manufacturing logistic templates for cell therapy [1,4]. To date, however, there are no authorized tissue-engineered products (TEPs) of parenchymal bioartificial organs or parenchymal tissue [5,6,7]. Other TEPs have already been authorized as advanced therapy medicinal products (ATMPs) by the European Medicines Agency (EMA) and the United States Food and Drug Administration (FDA) [5,6,7]. The small number of authorized TEPs includes products such as keratinocyte-containing scaffold sheets for traumatic burns, chondrocyte spheroids for cartilage defect regeneration, and autologous stem cells for corneal defect regeneration [5,6,7,8,9,10,11,12]. In contrast to such bioengineered bradytrophic and almost completely avascular bioartificial tissues, the scaled generation of solid parenchymal tissue with long-term viability and function still represents a major challenge in tissue engineering [4,13,14,15,16,17]. Based on existing study evidence, it is considered that constructs with dimensions of more than 100–200 µm need an endogenous vascular perfusion system to provide oxygen and nutrient supply and discard metabolic byproducts [18,19,20,21]. In parenchymal organ replacement therapy, all constructs need to be scaled to dimensions that need endogenous perfusion systems to achieve the necessary number of cells [15,18,21,22]. Thus, a major limitation central to any cell therapy approach is the establishment of perfusing vascularization in a TEP that supports cell survival, integration, and function [4,7,13,15,22,23]. At this moment, the objective is twofold: (i) acceleration of the process towards an anastomosis between bioartificial graft and host vasculature and (ii) the establishment of sufficient intragraft vascular density. The extensive vascularization of bioartificial tissue is a prerequisite for the long-term function of most transplanted cells [23]. Technological advancements such as three-dimensional (3D) bioprinting have led to intensified research interest in this interdisciplinary field [7]. Bioprinting enables the reproducible creation of 3D hierarchical tissues by the precise deposition of bioinks [4,13,14,21,22,24]. Bioinks are comprised of living cells, matrix compounds, signaling molecules, and logistic support materials [4,24] (Figure 1a). This broad definition includes simple monomaterial encapsulation systems for a single cell type but can extend to complex formulations of various materials combined with a variety of cell types and signaling molecules in a multibioink construct. The functionality of the desired bioartificial tissue can rely on one or more cell types, hereinafter called effector cells. Most commonly, biomaterials utilized as a support material in bioinks are hydrogels [21,24] (Figure 1a).

This review outlines bioink and bioprinting strategies for vascularization in an end-to-end manufacturing process. We will highlight concepts for the fabrication and integration of macro- and microvascularization. The intention is to allocate concepts to the respective stages—before, during, and after bioprinting—in the tissue fabrication process. Additional adjuvant processes will be discussed in this context. Moreover, we attempt to consolidate evidence from various experimental approaches by providing an integrated concept for bioprinting of vascularized parenchymal tissue.

### 1.1. Highlights

A broad overview of pre-, intra- and postprinting strategies for the biofabrication of vascularized parenchymal tissues in an end-to-end process based on state-of-the-art study evidence.

A review of bioink development strategies and material deposition strategies to bioprint vascularized tissue.

A discussion of an exemplary manufacturing process model for a fabrication and decision model for bioink development for future tissue-engineered products.

### 1.2. Outlook

The bioprinting of vascularized tissue is challenged especially with regards to producing small vascular networks and capillary-like structures. The integration of signaling molecules promoting vasculogenesis and angiogenesis, as well as cellular self-assembly strategies, might address limitations.

The combination of different material deposition techniques in different stages of the bioprinting process might facilitate the production of smaller, more natural vascular sturctures in biofabricated tissues.

Computer-aided tissue engineering by means of vascular tree modeling, in silico experiments, and flow simulation might help avoid trial-and-error approaches in data-driven bioconvergence research.

The combination of different material deposition techniques in different stages of the bioprinting process might facilitate the production of smaller, more natural vascular sturctures in biofabricated tissues.

## 2. Methodology

A literature survey was conducted in the PubMed/MEDLINE database to identify pertinent publications about bioink and bioprinting strategies for the vascularization of bioartificial tissue. ((bioprinting[Title/Abstract]) OR (3d-printing[Title/Abstract]) OR (bioink*[Title/Abstract]) AND ((vascular*[Title/Abstract]) OR (vessel[Title/Abstract])) was used as a search term. Study evidence published in the last 5 years and English language articles were focused. The last database access was carried out 26 June 2022. Based on these initially identified studies, extensive cross-referencing was performed. High-impact journals relevant to the field of research were searched to find additional study evidence.

## 3. Bioprinting Technology: A Brief Overview

Bioprinting is a broad term to describe the primarily layer-by-layer deposition of biocompatible or biodegradable materials together with integrated cells and therapy agents [13,23]. Although not the focus of this review, a brief introduction to state-of-the-art bioprinting technologies is provided to aid the understanding of specific bioink strategies and their relation to vascularization processes (Figure 1b–d). Each bioprinting modality has distinct requirements for the materials to be used [13,14]. Therefore, bioink and manufacturing techniques need to be attuned to each other. Primarily, bioprinting methods include jet-based drop-on-demand bioprinting, microextrusion bioprinting, and laser-assisted bioprinting techniques (Figure 1b–d).

### 3.1. Jet-Based Drop-on-Demand Bioprinting Inspired by Inkjet Printing

Inkjet printers are commonly used in a nonbiological context to deposit ink in a 2D fashion on paper. The first inkjet bioprinters were thus modified commercial inkjet printers with bioink replacing standard ink in the print cartridges [25]. An additional *z*-axis was introduced by a vertically adjustable print bed [25]. The specific printing features of this technology are similar to conventional 2D inkjet printing. Low-viscosity bioinks can be deposited at high speed and with high precision [13,14,23]. The diameter of the ink droplets ranges from 10 to 150 µm [1,20]. Droplet ejection is controlled via thermal or acoustic (piezoelectric) forces [25] (Figure 1b). Thus, bioink formulations for inkjet bioprinting must possess very low viscosities (<10 mPa s [21]) and require immediate, extensive layer-by-layer crosslinking to form 3D structures [15]. This requirement generally limits the spectrum of materials eligible for this technology [13]. The requirement of low viscosity also limits the maximum cell density in bioinks for inkjet printing [13]. However, the potential of droplet-based jetting can be significantly improved by the use of microvalves. The ability to actively open and close the valve nozzle means that bioinks can be subjected to a specific pneumatic pressure. In this way, the range of printable bioink viscosities can be significantly increased (up to 15 Pa s). At the same time, the survival rate of printed cells can be increased to up to 96% by selecting suitable nozzle geometries [26]. Harnessing these advances, artery-like structures with a wall thickness of 425 µm and diameter of 1 mm could be bioprinted [14]. The droplet-based deposition, however, can lead to uneven, edged shapes of vessel walls that may result in increased thrombogenicity in vivo [27]. Recently, Solis et al. reported beneficial effects for vascularization by thermal inkjet bioprinting inducing a temperature-based overexpression of proangiogenic signaling molecules [28].

### 3.2. Microextrusion-Based Bioprinting Inspired by Fused-Deposition Modeling

Microextrusion-based bioprinting, also called extrusion-based bioprinting, is inspired by non-biological 3D printers for the additive manufacturing of, for example, polymers.

3D-printing by fused-deposition modeling is a fast and cost-effective method for printing various materials [29]. The readaption of printers for bioprinting is not far-fetched. Although the high temperatures used in conventional 3D-printing are not suitable for bioprinting, by modification, conventional 3D-printers can be cost-effective microextrusion-based bioprinters [29]. Removal of the heating element and equipping the printer with a syringe pump system allows the deposition of bioink formulations [29,30]. Like polymers in fused-deposition 3D printing, bioink is extruded in the form of a continuous filament rather than as single droplets (Figure 1c). In layer-by-layer fashion, 3D constructs are built by the extrusion of bioinks from one or more print heads, the previous layer being the foundation for the next printed layer [21]. Bioinks are extruded by either pneumatic or mechanical forces (Figure 1c). Thus, compared to inkjet bioprinting, microextrusion enables the bioprinting of viscous formulations and higher cell densities [13,17]. However, viscous material deposition can also cause higher shear stress with a negative impact on cell viability [16,21,23,31,32]. Crosslinking can be achieved chemically, thermally, by ultraviolet (UV) light, or based on shear-thinning properties of the bioink. Recently, the development of in situ crosslinking technology for nonviscous bioinks was reported as a viability-conserving method in microextrusion [32]. Depending on the nozzle size, resolutions similar to those in inkjet printing are achieved, mostly at the cost of prolonged print times. From a technological perspective, a print resolution of up to 10 µm is possible; however, depending on the composition of the bioink and cellular material, this is not feasible. Generally, the lowest resolution of such bioprinting platforms is around 100 µm [21]. Microextrusion can be used to create vascular structures, as cells may be able to migrate within the construct and self-assemble [23]. Experimental direct and indirect bioprinting of vessel-like channel structures is mostly performed using extrusion-based bioprinting and is elaborated in the following sections.

### 3.3. Laser-Assisted Bioprinting

An as-yet less common and more costly technique is laser-assisted bioprinting. Pulsed laser beams induce the forward transfer of a biological specimen from a donor ribbon that contains an energy-absorbing layer onto a receiving substrate [13] (Figure 1d). This bioprinting modality is characterized by its excellent resolution [14,21]. High-resolution tubular capillary structures of 10 µm diameter can be fabricated [33]. Bioinks with cell density of up to 95% or highly viscous support materials (up to 1 Pa s) can be processed, because this nozzle-free technology avoids clogging of cells and materials [13,33]. Up to 10^8^ cells/mL can be deposited in a single-cell droplet resolution of up to 5 kHz and printing speeds of up to 1600 mm/s [33,34]. A drawback of laser-assisted bioprinting is the complex preparation of donor ribbons, especially for multimaterial, multicellular experiments [13]. The impact of laser beams on cell function and integrity requires further investigation [23,35]. Scaling up to manufacture large tissue specimens or structures resembling the hierarchical vascular tree may be difficult, and to date, there is no evidence for application of this technology to fabricate large-scale constructs [13,21]. With regard to vascularization strategies, it is crucial to consider proangiogenic features of both the bioink and the printing substrate.

## 4. Vascularization in Bioprinted Tissue: Different Approaches to Macro- and Microvascularization

**Figure 2:**
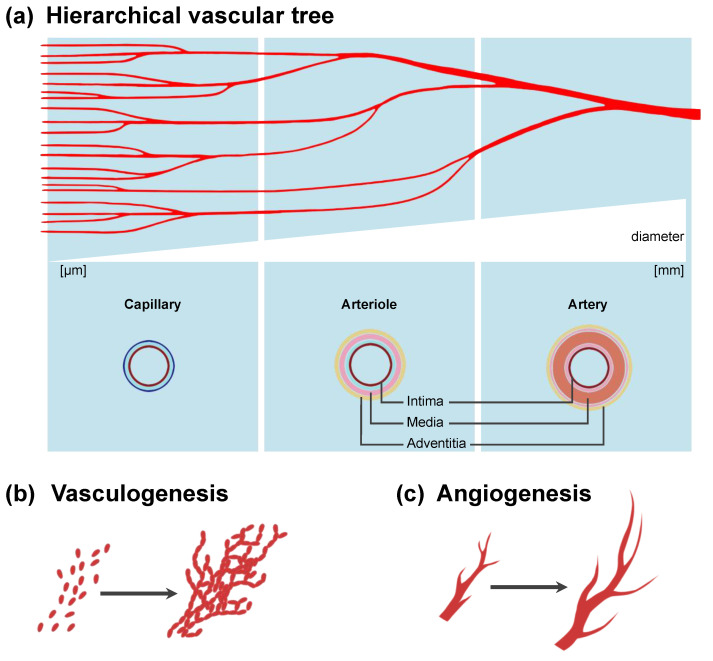
Overview of hierarchical vascular tree and vascular development mechanisms. (**a**) Partial representation of arterial vascular tree with dichotomous branching. Arteries and their smaller downstream branches, the arterioles, present a layered wall structure. Generally, the media containing smooth muscle cells is more pronounced in arterial vessels. Via the arterioles, blood is transported into the capillary bed, capable of gas and nutrient exchange. Capillaries consist of a single endothelial layer enabling permeability. (**b**) The mechanism of vasculogenesis describes the formation of a primitive vascular network by endothelial progenitor cells, usually during embryogenesis. (**c**) Angiogenesis describes a growth of vessels from the existing vasculature, e.g., by sprouting. Angiogenesis can be a physiological or pathological process.

In nature, the macrovasculature and the microvasculature differ in both structural composition and function [21,36] (Figure 2a). For the future bioprinting of parenchymal tissue with vascular trees, both systems need to be integrated into the manufacturing process. Macrovascular structures are required for anastomosis with the host circulation, whereas a high-density microvascular network is necessary to enable the transfer of oxygen and nutrients to the tissue. Artificial macrovascular arteries and veins have very limited transport and pressure-regulating functions [21]. The walls of arterial and venous vessels comprise three layers: (i) the inner tunica intima, with an endothelial lining; (ii) the tunica media, with smooth muscle cells; and (iii) the outer tunica adventitia, containing loose connective tissue and fibroblasts [21,36] (Figure 2a). Besides the geometric considerations, bioartificial vascular structures need to maintain resistance to thrombogenicity, immunogenicity, inflammation, and foreign-body response [36]. The response of a tissue to bioprinted grafts is modulated by its chemical, physical, and mechanical properties. To date, bioprinted macrovascular structures lack mechanical integrity due to insufficient crosslinking of the mature extracellular matrix (ECM) [36,37]. Bioartificial macrovascular medical products such as the poly(glycolic acid)-based, decellularized vessel Humacyte^®^ are currently in phase II clinical trials for arteriovenous fistula creation in selected patients [38]. This system has been demonstrated to be noninferior to synthetic vascular grafts [38]. However, although the engineered vessel has a diameter of 6 mm, primary patency rates of 63% at 6 months and 28% at 12 months indicate the need for improvements [38]. Cell-containing bioprinted products are yet to be developed [39]. On the other hand, microvascular capillaries enable gas exchange and protein and substrate transport to perivascular tissue [21,40]. The diameters of small vessels such as arterioles, venules, and especially capillaries are in the small submillimeter range (Figure 2a). While the biofabrication of macrovessels has already been successful in vitro, the engineering of the microvasculature remains challenging. The direct or indirect printing of vascular structures cannot be completely scaled down to reproduce a capillary bed and is mostly limited to the manufacture of structures larger than 100 µm [39,41,42,43]. Thus, biomaterials, or rather bioinks themselves, may need to provide cues that promote vessel formation. Such self-assembly of a microvascular network can occur either by vasculogenesis (Figure 2b) or by angiogenesis (Figure 2c). The term vasculogenesis describes the completely new formation of a vascular structure and primitive vessel network, typically driven by endothelial progenitor cells (EPCs) in embryonic development [21,44] (Figure 2b). However, vasculogenesis is not limited to prenatal development [45]. Angiogenesis refers to capillary network formation starting from a pre-existing vessel, e.g., by sprouting [21,33] (Figure 2c). Both mechanisms need to be considered in the bioprinting of parenchymal tissue.

## 5. Strategies before 3D Bioprinting: Material Properties and Bioink Formulation Can Alter Vascularization Processes

### 5.1. Physical Properties of Bioink and Geometry of Construct Can Positively Influence Vascularization

Among other factors, the mechanical and topographical requirements for bioink formulations have to be considered in the attempt to mimic nature and, thus, provide an optimal microenvironment for cell proliferation [42,46]. Pore size, the interconnectivity of pores, and geometry are essential parameters influencing the mass transfer of, for example, oxygen and glucose [4,23,47] (Table 1). After printing, the pore and surface geometry of bioink materials can promote and accelerate vascularization [4,23,47] with high porosity and extensive pore interconnectivity, e.g., introduced by the protease-sensitive degradation of support materials to enhance and accelerate intraconstruct vascularization [47,48,49] (Table 1). However, synthetic-polymer-based bioinks do not offer intrinsic interstitial space comparable to the natural ECM [48]. In 3D cell culture experiments, it was demonstrated that vascular invasion increases with pore size in poly(ethylene glycol) (PEG) hydrogels [48]. This bioink material showed limited cell and vessel invasion at pore sizes of 25–50 µm, in contrast to extensive vascularization at pore sizes of 50–100 µm and 100–150 µm [48]. Similarly, chitosan bioink was reported to promote neovascularization at pore sizes ~90 µm, whereas this process was absent at pore sizes of ~30 µm [49]. At the upper end, pore sizes of >400 µm did not trigger a greater extent of neovascularization [49]. It is worth noting that larger pore sizes not only increased the extent or density of neovascularization but also accelerated the vascularization process [48]. In the context of porosity and pore size tuning, it is important to consider inflammatory tissue layers as, to some extent, a sign of foreign-body response, because such tissue layers will decrease the effective pore size eligible for vascular invasion in vivo [48]. In synopsis, evidence suggests that there is a lower limit for pore size of ~30 µm under which vascular ingrowth and vessel formation is reduced. In contrast, there also seems to exist an upper limit of ~400 µm, where no increase in vascular ingrowth can be found at the expense of reduced graft volume.

In silico modeling for defined architectures can predict postimplantation viability and function in vivo [50]. Defining a construct geometry in silico is a process commonly required in all additive manufacturing technologies. Computer-aided design and in silico analysis of these geometries prior to the manufacturing process itself showed the potential to improve on trial-and-error experimental approaches [50,51] (Table 1). Previous studies on the bioprinting of insulin-producing constructs showed the potential of finite element analysis for simulation of diffusional processes of oxygen, glucose, and insulin outflow [50,51,52]. The advantage of such approaches was to define boundary conditions, e.g., the filament thickness of the extruded hydrogel and, thus, the maximum diffusion distance for effector cells to remain viable and functional [50] (Table 1). We previously showed that without a sufficiently uniform perfusion system throughout the construct, cells are more likely to populate the periphery of the bioprinted construct without uniform cell density [50]. Especially for highly metabolically active cells such as insulin-producing ß-cells, vascularization strategies are necessary [4].

### 5.2. Biochemical Properties of Bioink Can Positively Influence Vascularization

In nature, various cell types and the ECM form an organ-specific microenvironment [53,54]. Therefore, one paradigm in tissue engineering and the bioprinting of functional tissue is to resemble this natural microenvironment to maintain the physiological functionality of effector cells [4,53,54,55,56]. There are numerous studies on bioink formulations that specifically support effector cell viability and function [24]. In recent years, the market for commercially available bioinks has been growing [24]. ECM composition and structural binding motifs in bioinks can also affect vascularization within bioartificial tissue [40,46,47,57]. Bioinks, therefore, often contain biomaterials such as collagen and fibrin, which have been reported to support angiogenic growth due to their binding motifs [39,58,59,60] (Table 1). In addition, naturally derived materials such as hyaluronic acid, dextran, agarose, and gelatin, but also synthetic materials such as PEG, can be adapted to enable vascularization [47,57] (Table 1). Study evidence suggests enhanced integrin-mediated adherence of endothelial cells (ECs) by integration of arginine–glycine–aspartic acid sequencing (RGD) motifs [42,47] (Table 1). Such motifs are present in natural hydrogel materials such as collagen, gelatin, gelatin methacrylol, fibrin, and hyaluronic acid [55,58,61]. In alginate, agarose, and PEG hydrogels, such motifs are missing but can be added to the bioink formulation [42,62,63]. In a systematic investigation of the viability of human umbilical vein endothelial cells (HUVECs) in various hydrogels of standardized concentrations, Benning et al. demonstrated that fibrin, collagen 1, Matrigel, and gelatin hydrogels maintain the viability of integrated HUVECs and enable the attachment of cells [64]. In contrast, alginate and agarose do not enable cells to attach, resulting in inferior cell viability [64]. The same effects were found for cell proliferation, with the latter hydrogels inhibiting the proliferation of ECs [64]. Interestingly, collagen, Matrigel, and gelatin hydrogels showed an inverse correlation of hydrogel concentration to cell proliferation, with lower concentrations enabling enhanced proliferation [64] (Table 1). EC sprouting for capillary formation was observed only in collagen, Matrigel, and fibrin hydrogels; it was not present in gelatin or alginate hydrogels [64]. Although collagen 1 seems to be an excellent support material in bioinks from a vascularization perspective, concentrations >3 mg/mL are reportedly too dense to allow capillary formation and sprouting [65]. Furthermore, due to slow gelation kinetics, extrusion- and droplet-based printability and shape fidelity are impeded [21,66] and might require other bioprinting techniques, such as laser-assisted bioprinting [67], or blending with other hydrogels [59,66]. Bioprinting using the decellularized ECM from vascular tissue as bioink has been reported to provide a better microenvironment for EC viability and proliferation than type 1 collagen bioink [68]. Thorough processing of nonautologous ECM specimens is crucial in order to avoid pathogen transfer and host immune response [21]. In addition to ECM composition, bioinks can also be tuned by proangiogenic signaling molecules [23]. The most prominent signaling molecule reported to be integrated into bioinks is vascular endothelial growth factor (VEGF) [4]. Farina et al. reported a dose-dependent increase in vessel density after loading of bioinks with VEGF (0.5 µg/mL vs. 5.0 µg/mL) [69] (Table 1). However, high VEGF dosage (5.0 µg/mL) led to a pathological alteration of vessel structures [69]. In a two-component microencapsulation approach, Weaver et al. demonstrated the applicability of vasculogenic, degradable hydrogel materials to promote vascularization [70]. The coating of a bioink core with a VEGF-containing degradable outer layer (10.0 µg/mL) showed enhanced vascular network formation in vivo [70]. Enhanced surface vessel density around the bioink led to increased viability of the effector cells in the inner bioink core [70]. Proangiogenic signaling may be especially helpful for microvascularization and capillary sprouting [47]. After direct bioprinting of relatively large vessels, integration of proangiogenic molecules (50 ng/mL VEGF, 30 ng/mL fibroblast growth factor 2) into the surrounding bioink microenvironment led to angiogenesis [47]. After 7 days in vivo, incorporation of VEGF (100 ng/mL) into a porous collagen 1 construct led to vessel penetration depths of ~200 µm [71]. In a more versatile experimental model, Song et al. demonstrated microvascular sprouting from a bioartificial vessel-like structure 300 µm through a support hydrogel in 3 days by application of a proangiogenic gradient (VEGF 100 ng/mL, phorbol-12-myristate-13-acetate 600 ng/mL, and sphingosine-1-phosphate 500 nM) [47,72]. However, angiogenesis 400 µm into a collagen matrix surrounding a bioprinted vascular channel has also been demonstrated without any growth factor supplementation [27]. Comparison of study evidence and definition of the necessity and optimal dosage of proangiogenic signals is impeded by several confounding parameters such as hydrogel composition, density, and culture conditions; standardized studies are required. In this context, it is worth noting the existence of bioink strategies attempting to bypass the initial critical period before the sufficient self-assembly of a microvascular network. There are bioink materials or composites specifically developed to address the insufficient oxygenation of effector cells. OxySite^®^ is a hydrolytically activated oxygen-producing biomaterial consisting of polydimethylsiloxane and calcium peroxide [52,73,74] (Table 1). Although hybrid constructs with OxySite^®^ showed improved viability and function in vivo [74], the manufacturing process was mostly manual and has yet not been scaled up to human application. Incorporation of OxySite^®^ microbeads [75] into bioinks and application in bioprinting technology might be a focus for future studies.

In addition to the cell biological functionality, the form stability of applied bioinks should be taken into consideration [76]. This applies not only to the biofabrication process itself, but also to the subsequent tissue maturation phase. Especially for the latter, cell-driven bioink contraction plays a vital role [77]. Previous studies have shown, for example, that ECM-derived gels (e.g., collagen or fibrin), which are particularly well suited for vascularization, contract strongly during prolonged culture and, thus, impede shape fidelity [78,79]. Their high stress-relaxation capacity and high degree of adhesion-ligand-binding motifs, which promote cell proliferation and microenvironmental remodulation, are potential drivers of this effect [80]. Modulation of the stress relaxation response of bioinks, e.g., by blending with polysaccharide-based hydrogels (e.g., agarose or alginate), was shown to prevent excessive gel contraction without limiting biofunctionality [78].

### 5.3. Cellular Composition in Bioink Can Positively Influence Vascularization

Above, we have described the ability of the bioink-supporting material itself and alterations of such carrier material by the addition of signaling molecules to enhance the vascularization of bioprinted tissue. Furthermore, integration of living cells into the bioink has been found to influence vascularization properties [4]. Besides effector cells for respective functions of the manufactured tissue, additional cell types have been studied with regard to the possible promotion of vascularization [15,50,81]. The inner lining of vessels is built by ECs. Confluent endothelial lining can be detected by VE-cadherin staining [14,27,47]. Thus, it seems apparent that ECs and their progenitors are among the most investigated cell types regarding vascularization of the construct [82]. Such additional cell types can be directly integrated in a bioink, housing the effector cells in a form of coculture [15,50]. Moreover, several bioinks consisting of different supporting materials specifically adapted to the respective cell compositions can be applied in combination on a bioprinting platform [14,46]. HUVECs have been extensively studied for application in biofabrication [14,21,27,33,39,46,47,50,59,63,83,84,85,86,87,88,89]. Functional vessel endothelium produces nitric oxide, thrombomodulin, and tissue plasminogen activator, all inhibitors of thrombus formation [36,40]. It has been shown in vitro that coating of bioink constructs with HUVECs diminishes thrombogenicity in the whole circulatory system [18] (Table 1). HUVECs are derived from larger umbilical veins. Although there is no evidence from bioprinting studies, their potential to build microvascular networks is a topic of heated discussion [21]. Interestingly, although there is extensive evidence on specific EC subtypes in the vascular system of organs, and extensive knowledge about differences between ECs depending on the type of vessel, these factors have rarely been considered in experimental bioprinting approaches [21,40,42,82,90,91]. Organ-specific ECs such as human liver sinusoidal microvascular ECs [92], dermal microvascular ECs [81], and renal proximal tubule ECs [93] might be worthy of more extensive exploration, as they present the prospect of a superior microenvironment similar to nature [21,42,90] (Table 1). Some strategies have integrated EPCs instead of mature cells [94,95]. Moreover, substantial effort has been invested in the development of strategies for endothelial and perivascular cell differentiation from human-induced pluripotent stem cells (hiPSCs) [46,91,96,97,98] (Table 1). This nearly limitless autologous cell source can be utilized for patient-specific tissue engineering [46,98]. In addition to ECs, pericytes, smooth muscle cells, and fibroblasts participate in vessel network formation [14,17,36,42,85,86,89,96] (Table 1). Thus, there is study evidence that coculture with endothelial-stabilizing cells enhances vessel network formation, e.g., by growth factor secretion and direct cell–cell interactions [33,42,60,63,89,99]. Mesenchymal stem cells, sometimes also referred to as medicinal signaling cells [100], have been widely used in tissue-engineering strategies with the intention of promoting functional vascularization by VEGF secretion [17,39,42,58,59,101]. Additionally, mesenchymal stem cells have the potential to differentiate to smooth muscle cells, thereby further resembling the natural vascular cellular composition [42,84,100] (Table 1). There is little evidence of bioink strategies for bioprinting vascularized tissue that involve cocultures or the combined application of several proangiogenic cell types. Studies on the effect of combinations of proangiogenic cell types and their respective quantitative proportion might be beneficial.

**Table 1:** Overview: Bioink properties and composition can influence vascularization.

**Physical properties and geometry**	**Porosity**	
	High porosity and pore interconnectivity increases mass transfer	[4,23,47]
	**Pore size**	
	Larger pore size leads to increased neovascularization and accelerated vascularization process	[48]
	**Architecture (in silico modeling)**	
	Adaption of model to boundary conditions such as maximum diffusion distance of nutrient flow might enable uniform perfusion	[50,51,52]
**Biochemical properties**	**Structural binding motifs**	
	RGD motifs promote endothelial cell adherence	[42,47]
	Motifs can be offered naturally or added to bioink formulations	[42,55,58,61,62,63]
	**Hydrogel concentration**	
	Lower collagen, Matrigel^®^ and gelatin concentrations enable enhanced proliferation	[64]
	**Proangiogenic signaling molecules**	
	VEGF addition causes dose-dependent increase in vessel formation	[23,69]
	**Oxygen-producing bioinks**	
	Critical oxygen-supply before self-assembly of microvasculature OxySite^®^ can address locally insufficient oxygenation	[52,73,74]
**Cellular composition**	**Endothelial cell coating**	[18]
	Diminished thrombogenicity	
	**Organ-specific endothelial cell sources**	[77,88,89]
	More natural microenvironment	[21,42,90]
	**Human induced pluripotent stem cells (hiPSCs)**	[46,91,96,97,98]
	Autologous cell source for patient-specific tissue engineering	[46,98]
	**Pericytes, smooth muscle cells and fibroblasts**	
	Endothelial-stabilizing cells for co-culture	[33,42,60,63,89,99]
	Growth factor secretion and cell–cell interactions promote vascularization	
	**Mesenchymal stem cells (MSCs)**	
	VEGF secretion promotes functional vascularization	[17,39,42,58,59,101]
	Differentiation into smooth muscle cells to resemble a natural cellular environment	[42,84,100]

## 6. Strategies during 3D Bioprinting: Modifications in Material Deposition Enable Fabrication of Vessel-like Networks

### 6.1. Application of Sacrificial Material and Sacrificial Writing into Functional Tissue for Vascular Network Integration

**Figure 3:**
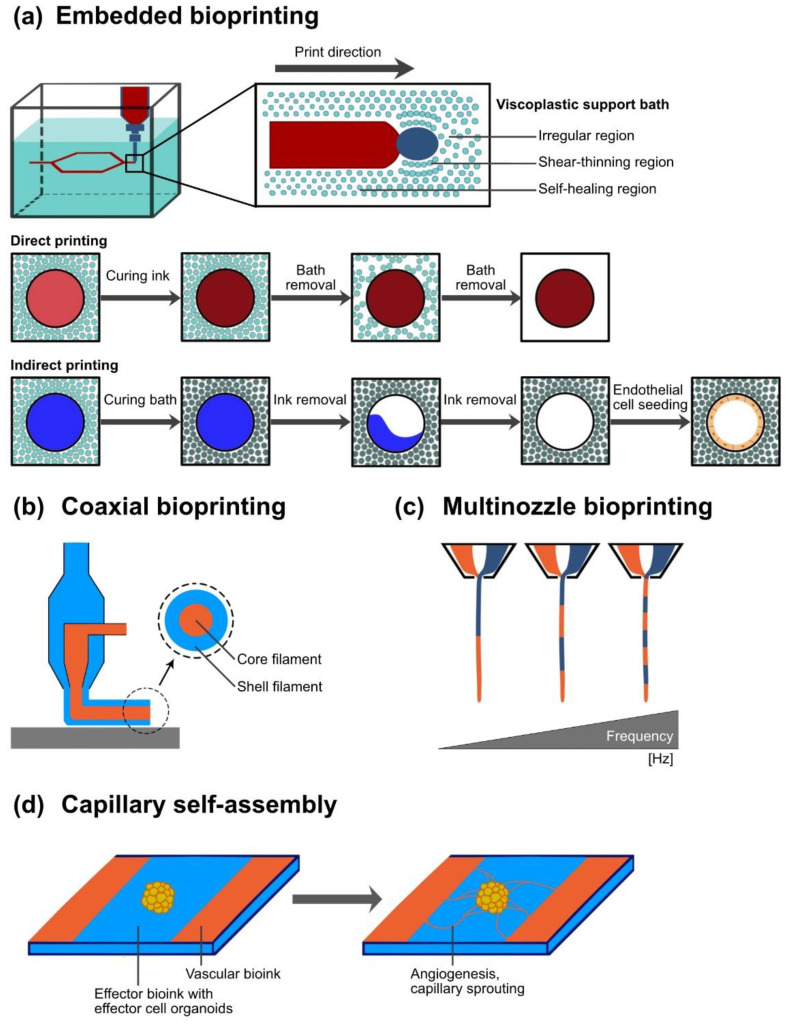
Bioprinting strategies to fabricate vascularization. (**a**) Embedded bioprinting into a (functional) viscoplastic support bath. Viscoplastic microsphere fluids present self-healing properties with fluid-like regions and shear-thinning effects directly along the moving microextrusion nozzle. Direct printing and subsequent in situ crosslinking of vascular bioink enables fabrication of free-form structures with high shape fidelity. Crosslinking can be initiated by the support bath, e.g., chemically. The microextrusion and subsequent dissolution of sacrificial bioinks after previous curing of the functional support bath can create hollow channel networks. Such networks can be perfused with endothelial cell solution for cell adherence and formation of an endothelial lining. (**b**) Coaxial microextrusion bioprinting using concentric nozzles enables simultaneous printing with different bioinks with a core–shell or layered cross-section. (**c**) Multinozzle bioprinting technology with fast high-frequency switching can fabricate continuous filaments from multiple materials. (**d**) Self-assembly of capillaries from bioprinted vessel-like structures. The cultivation of bioprinted tissue constructs can provide an environment that further enhances vascularization by self-assembly. Therefore, relevant angiogenic signals might need to be integrated in bioinks before printing.

In recent years, sacrificial or fugitive inks have been used especially for integrating vessel-like networks into bioprinted tissue constructs (Figure 3a, an overview about sacrificial bioprinting is provided in Table 2). Sacrificial inks can be bioprinted to shape a template that serves as ‘negative’. Gelatin [14,22,27,39,47,63,83], alginate [87,102,103], Pluronic^®^ F127 [68,85,86,104,105,106,107], agarose [108], poly(vinyl alcohol) [109], and carbohydrate mixtures [43,88] can serve as sacrificial inks. In a next step, sacrificial templates are embedded in low-viscosity bioinks containing effector cells, which then can be adequately cured [110]. After sufficient crosslinking, the sacrificial materials are removed [110]. Removal can be accomplished by dissolution with a solvent [14,47,85,86,103,106,110,111], temperature regulation [14,27,46,83,104,105,106,110], or pH regulation [58,85], leaving a microfluidic network of patterned microchannels or even vascular tree-like channels in the construct [22,47,83,87,110]. Perfusion of such a network with ECs and their subsequent partial adherence has the potential to result in coating of the inner wall [27,47,63,83,87,105,111] (Figure 3a). Other studies reported a method in which ECs (8–20 × 10^6^/mL) were directly integrated into the sacrificial gelatin core [14,27,46]. Instead of direct perfusion, the liquified sacrificial ink was cultivated for 2–4 h to allow EC attachment, resulting in a confluent layer after 4 days of cultivation [14]. Luminal diameters below 10 µm are found in the capillary bed [112]. However, even though such small-scale network resolutions might be possible by application of other technologies, EC perfusion resulted in clogging [113]. The smallest fabricated perfusable microvessel had a diameter of 50 µm [113]. EC coating depends on many parameters, such as the surface roughness and biocompatibility of the effector bioink, cell density, seeding time, and culture conditions [68]. After perfusion and adherence of ECs, a confluent cell lining is found on smooth wall surfaces [47]. A smooth endothelial lining is promoted by perfusion flow culture rather than static culture [27]. A special use of sacrificial material can be found in embedded bioprinting platforms (Figure 3a, an overview is provided in Table 2). Especially soft hydrogel bioinks (<100 kPa) are subject to gravity-induced shape loss when printed without support [21]. The use of support baths, which mitigate the influence of gravity on the bioinks’ dimensional stability, offers a potential solution. For instance, the Feinberg group introduced the technique of freeform reversible embedding of suspended hydrogels (FRESH) in several publications in 2015 [114]. The basic idea is to use viscoelastic support baths of sacrificial material (e.g., gelatin microspheres) as a ubiquitously supporting three-dimensional print bed [114] (Figure 3a). The support bath material needs to possess the yield stress behavior of Bingham plastic fluids to allow the extruder to move through the gel without resistance, while at the same time, the support material solidifies around the extruded bioink to alleviate gravity-based shape loss. The developed system supports the shape fidelity of the extruded bioinks until the curing process is finished [114] (Figure 3a). After complete curation, the support bath can be liquified and completely removed from the construct [71,114] (Figure 3a). Resolution of bioink deposition by embedded bioprinting is dependent, among other factors, on microsphere homogeneity and size [114]. Larger and heterogeneous microspheres in the support bath will restrict the resolution to larger-diameter filaments with variable morphology [71,114,115]. It was reported that bioink resolutions of 20 µm diameter can be achieved by means of this system [71]. In addition to beneficial effects on the shape fidelity of bioink structures, the support bath can contain cell culture medium and, for example, growth factor supplements to prevent dehydration and to provide nutrients or oxygen during the printing process for the maintenance of cell viability. For example, perfluorocarbon-based support baths have been shown to not only provide mechanical support but also enable the exchange of respiratory gases, essential during prolonged printing processes [116]. The support bath itself can initiate a curing process by ionic, enzymatic, UV-, or pH-dependent gelation [71,87] (Figure 3a). A comprehensive overview on bioink and support bath materials applicable for embedded bioprinting has been provided by Shiwarski et al. [115]. Bioprinting a combination of several bioinks in one support bath is possible. However, the gelation mechanism initiated by the support material must be suitable for all embedded bioinks [115], so free combination is not possible. Obviously, the cross-linking mechanism must not be effective on the support bath itself. The described approach was used for direct bioprinting of patent, perfusable vascular structures with an inner diameter of 1.4 mm and a wall thickness of 300 µm [71]. Therefore, a bioink of collagen type I and a myoblastic cell line were applied [71]. Furthermore, more complex, perfusable vascular trees were directly bioprinted based on an MRI-templated multiscale vasculature mesh [71]. The mesh included branched vessels with a resolution of up to 100 µm [71]. Extrusion-based bioprinting enabled three-generation branching with a minimal channel diameter of 30 µm [107]. Bioprinting of small-scale microvasculature (e.g., capillaries of 5 µm diameter) is technically not possible by means of the direct extrusion-based method. Lee et al. described the direct incorporation of the FRESH support bath microspheres themselves into the construct to promote neovascularization in vivo by increased porosity [71]. In this context, another approach is to bioprint sacrificial microchannels in effector bioink baths (Figure 3a, an overview is provided in Table 2). Shear-thinning, self-healing bioink formulations allow printing of hydrogel in hydrogel [31,47,87,107,117]. Such viscoplastic bioinks that are tunable depending on shear force can be manufactured using supramolecular encapsulation systems [31,47,117]. The integration of perfusable, vessel-like channels in bioartificial tissue (or effector bioink) is referred to as sacrificial writing into functional tissue (SWIFT) [83]. A bulk volume of ~400,000 merged organoids developed separately was compacted in a gel-based 3D culture with high cellular density similar to that of natural tissue—exceeding the cell density of bioinks eligible for extrusion-based bioprinting [83]. A microchannel system is introduced into this effector cell gel by extrusion of sacrificial bioink [83,87]. Skylar-Scott et al. adapted this concept, initially studied in avascular, viscoplastic, ‘self-healing’ hydrogels, for bioprinting vessel-like structures directly in functional tissue blocks [83]. The embedded bioprinting of gelatin sacrificial bioink (5% w/v) resulted in a continuous and interconnected channel system within the bulk gel [83] (Figure 3a). During printing, it is essential for the sacrificial bioink to have a shear yield stress significantly higher than the embedding volume to ensure high shape fidelity of the channel structures [83]. In this study, channel structures with a diameter range of 1 mm to 400 µm were achieved. Smaller channel structures could not be achieved due to the inherent characteristics of the embedding bulk volumes with organoid structures of ~200 µm in diameter [83]. Besides embedded bioprinting of sacrificial bioink, the authors also demonstrated the applicability of the concept for effector cell-containing bioinks, thus expanding the potential for the precise spatial deposition of bioinks within the construct [83].

### 6.2. Coaxial Printing Technology for Vessel-Like Structure Fabrication

McGuigan and Sefton developed a modular concept for the manufacture of an interconnected, random-pattern perfusion system for bioink modules [18]. In their concept, small cell-containing collagen tubes were manually manufactured and, subsequently, each module was coated with HUVECs [18]. The HUVEC-coated tubes were arranged in an external tubular housing. The authors demonstrated the feasibility of a perfusable, random-pattern system with vessel-like endothelial lining. However, a tree-like vascular network could not be generated and bioprinting on the capillary scale was not possible [18]. Additionally, the construct lacked a macrovascular connection anchor. The natural vascular tree dynamically alters flow rates and, thus, the pressure differences and shear stresses at the endothelial lining along its branches [40]. Exceeding the shear stress thresholds of ECs (5–50 dynes/cm^3^ depending on vascular origin [118]) can lead to phenotypical remodeling with a subsequent alteration of physical and biochemical reactions to hemodynamic factors [18]. Coaxial bioprinting has the potential to address these limitations (an overview is provided in Table 2). In the context of this review, coaxial printing is defined as the simultaneous, separate extrusion of different bioinks via one print head, usually by concentric extrusion nozzles (Figure 3b). It offers precise control over the spatial distribution of multiple cell types or signaling molecules, thus allowing the creation of gradients within and around the bioprinted construct. Liu et al. utilized coaxial bioprinting technology for the one-step manufacturing of coated filaments by coaxial extrusion of a bioink core containing effector cell clusters and a bioink shell containing vascular progenitor cells [119]. Two concentric nozzles with an inner diameter of 450 ± 15 µm for the core filament and 1045 ± 25 µm for the shell filament were mounted on one cantilever print head, but were accessed and served by two independent mechanical extrusion systems [119]. The core–shell ratio and the filament thickness, respectively, can be regulated by printing speed and extrusion pressure in addition to the static relation of the concentric nozzles [85,119,120]. Direct bioprinting of vessel-like luminal structures with EC-containing shells of different diameters was reported in several studies [68,84]. Gao et al. bioprinted vessel-like tubes using a coaxial bioprinting setup [68]. By coaxial extrusion, a sacrificial core of the triblock copolymer polyethylene–polypropylene–polyethylene Pluronic^®^ F127, and Ca^2+^ was manufactured simultaneously with a bioink shell of decellularized ECM, alginate, and HUVEC [68]. Ca^2+^ enabled the direct crosslinking of alginate, and the subsequent thermal gelation of collagen portions stabilized the construct to be free-standing even after the dissolution of Pluronic. The authors demonstrated the feasibility of the concept for the printing of hollow vessel-like tubes with a wall thickness of 49 ± 21 µm and inner diameters of 853 ± 18 µm, 507 ± 26 µm, and 247 ± 31 µm [68]. The presence of an intact endothelial lining without overproliferation was demonstrated after 7 days in vitro [68] and may lead to faster constitution of an intact confluent endothelial lining than with perfusion-based cell-coating systems [83,105]. However, the vessel walls were vulnerable due to either low wall thickness or limited representation of vascular cell types, thus inhibiting pump-based perfusion. Sacrificial core filaments stabilize the otherwise hollow construct until sufficient curing of the material has been achieved [111]. Coaxial bioprinting platforms have also been modified to crosslink shell structures, mimicking the vascular wall, by the simultaneous extrusion of ionic crosslinking agents instead of any sacrificial core filament or by in situ crosslinking [16,32,84,120]. One technological shortcoming of the coaxial extrusion is the necessity of generating a continuous filament flow to avoid leaks. Thus, the generation of branching requires further technological advances [16]. In summary, this bioprinting technology may elicit the generation of at least partially bioartificial vascular trees. Coaxial bioprinting approaches offer the possibility of splitting the typically single stream of bioink into multiple bioink filaments extruded simultaneously (Figure 3b). Different bioink loadings of split channels and different extrusion systems broaden the flexibility of construct design and precise cell deposition [111]. The cross-section complexity is limited to the arrangement of extrusion nozzles (concentric or parallel) (Figure 3b). Flexibility might be enhanced by the rapid prototyping and manufacturing of customized coaxial extrusion nozzles [111]. The layered wall of vascular structures has physiological significance. Coaxial extrusion is capable of manufacturing such layered tubular structures, mimicking nature [85,121]. Circumferential multichannel coaxial extrusion systems were used to manufacture bioartificial luminal vascular tissue with an inner endothelial lining and an adjacent outer smooth-muscle-cell layer [85,121]. Bosch-Rué et al. described a triplex coaxial nozzle extrusion system to print layered, luminal, vessel-like structures with nozzle diameters of 570 µm for the inner sacrificial Pluronic core, 1.15 mm for the middle endothelial layer and 1.83 mm for the outer muscle layer [85]. Collagen type I in a high concentration of 20 mg/mL as bioink support material for HUVECs and smooth muscle cells, respectively, had sufficient shape fidelity to enable the omission of a sacrificial support bath during printing while maintaining a cell viability of 85.8% at 24 h after printing [85]. Even though a combined wall thickness of 200 µm for the endothelial and muscle layers was achieved, the maximum burst pressure of 620 mmHg and the maximum flow rate with shear stress of 10 dynes/cm^2^ were inferior to natural arteries [85].

Recent technological developments enable the manufacturing of more complex tissues by multimaterial, multinozzle bioprinting [122,123] (Figure 3c). Versatile extrusion systems for bioprinting with fast switching of different bioink-containing nozzles generate one continuous filament outflow of heterogenous, segmental bioink composition [122] (Figure 3c). By application of this sophisticated extrusion system, the minimal segment of a respective bioink along the filament was equal to the diameter (D) of the nozzle, thus enabling a print resolution of D^3^.

**Table 2:** Overview: Bioprinting strategies to fabricate vascularization.

	Explanation	Advantages	Disadvantages	Example Materials	
**Sacrificial bioprinting**	Deposition of a material, that can be removed in a subsequent stage	Easy removal of sacrificial material High degree of geometrical freedom	Printed structure prone to drying out Limited to sacrificial materials removable under cytocompatible conditions	Natural and synthetic hydrogels: gelatin,	[14,22,27,39,47,63,83]
				agarose,	[108]
				and alginate	[87,102,103]
				Pluronic^®^ 127	[68,85,86,104,105,106,107]
				Thermoplastics: PVA	[109]
				Carbohydrate mixtures	[43,88]
**Sacrificial Writing**	Extrusion of sacrificial material into functional tissue or merged organoids, respectively	High cell density, exceeding capability of microextrusion bioprinting Native ECM secreted by organoids	Limited resolution of vessel diameter (400 µm)	Sacrificial gelatin	[83]
**Submerged bioprinting**	Bioinks containing cells are 3D-printed into a support bath (e.g., high-density liquids and hydrogel slurries)	Placement into support bath prevents printed structure from drying Mechanical support for shape fidelity and geometrical freedom	Large volume of surrounding matrix necessary Postprocessing (washing) can damage fine structures	Perfluorocarbon Hydrogel slurry:	[116]
				gelatin → FRESH	[71]
				agarose → CLASS	[124]
**Coaxial bioprinting**	Simultaneous printing of at least two materials by same cantilever axis	Direct printing of vessel-like structures with core and shell Printing of layered vessel wall	Challenges in printing branched structures	Sacrificial materials Ionic crosslinking agents	[68] [16,32,84,120]

## 7. Adjuvant Strategies for Vascularization

### 7.1. Prevascularization of Transplantation Site to Accelerate Graft Function

**Figure 4:**
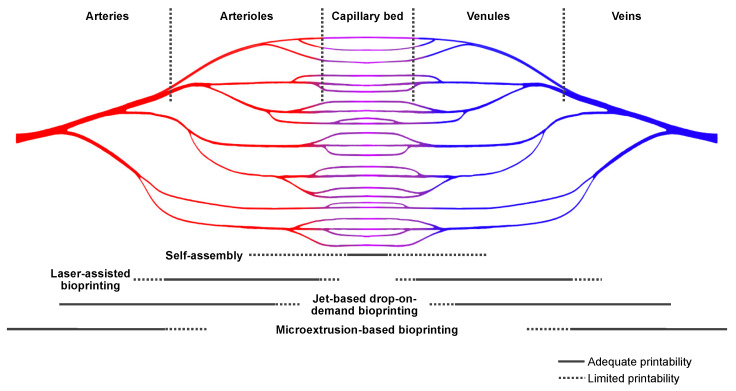
Bioprinting technologies categorized regarding their applicability for fabrication of macro- or microvascularization. Limited printability can be due to lack of resolution, low printing precision, and/or a long duration of the printing procedure. Small-scale capillaries cannot yet be fabricated by 3D-bioprinting technologies and are subject to self-assembly strategies.

Although versatile bioprinting strategies have been studied for the fabrication of vascular structures within a functional construct, the direct printing of capillary structures is still limited (Figure 4). Thus, postprinting strategies and cultivation of bioprinted constructs address augmentation and acceleration of self-assembly of capillary structures (Figure 3d and Figure 4). In addition to preprinting processes that focus, for example, on the integration of angiogenic cues in bioink formulations, there are also adjuvant postprinting strategies to promote vascularization. Several studies have proposed a two-stage approach enabling sufficient prevascularization prior to effector cell loading [15,69,125,126,127,128,129]. Prevascularization can be induced by in vivo implantation of a foreign body in the weeks leading up to implantation of the effector-cell-containing bioprinted construct. Local inflammatory and immune reactions cause enhanced angiogenesis [15]. CellPouch^TM^ is an implantable and retrievable device for transplantation and long-term housing of effector cells and tissue [128,129]. The device is implanted subcutaneously several weeks prior to effector cell loading into the device [128,129]. The prevascularized housing enabled successful grafting of thyroid tissue and pancreatic islets in vivo [128,129]. Transient implantation of a foreign body before effector cell implantation can avoid autoimmunity and foreign body response while maintaining angiogenic effects [130]. Furthermore, by means of microsurgical techniques, arterial–venous (AV) loops can be created in vivo. AV loops likewise induce angiogenesis at the respective site, while at the same time, they can serve as connection vessels between the graft and the host circulation [15]. Such approaches are intended to diminish an initial critical period after implantation in which the supply of nutrients to the effector cells relies solely on diffusional processes until vascularization occurs. Smink et al. performed the subcutaneous implantation of scaffolds into diabetic mice [126]. Four weeks after implantation, rat Langerhans islets were injected into predefined channels. However, the authors showed that even after prevascularization, restoration to normoglycemia took significantly longer in the scaffold group than in control animals that received direct implantation of islets under the kidney capsule [126]. In addition, a smaller number of islets was acceptable to restore normoglycemia in most animals of the kidney capsule group. Although this study showed successful local prevascularization and subsequent vascular network formation in the scaffold, the extent of vascularization seemed inferior to the nutrient supply found under the naturally vascularized kidney capsule [126]. Delayed graft function due to prolonged vessel maturation processes even after prevascularization was confirmed by other studies [69].

### 7.2. Generation of a Bioartificial Vascular Tree

**Figure 5:**
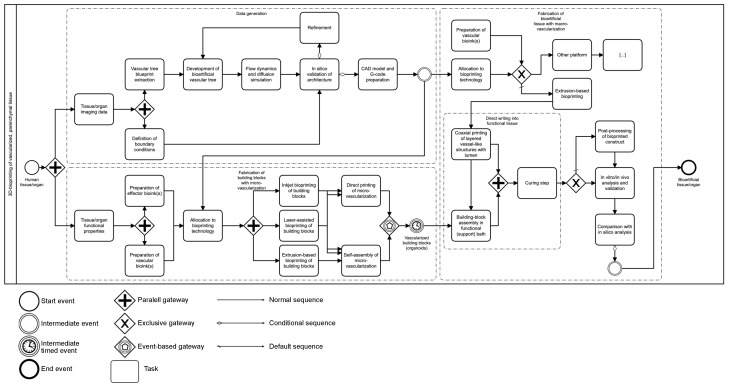
End-to-end manufacturing concept based on imaging data. Discussion of a concept for 3D bioprinting of vascularized parenchymal tissue. Process model for fabrication of vascularized parenchymal tissue potentially integrating several printing technologies in one fabrication process (business process model and notation [BPMN] 2.0).

Refined extrusion technology and bioprinting platforms allow the direct manufacture of vessel-like structures down to a diameter of 30 µm [22], or in the case of laser-assisted bioprinting, 10 µm [33]. In a previous study, it was shown that a scalable, branched, random-pattern channel network spanning several magnitudes in size could be bioprinted by taking advantage of the viscous fingering phenomenon [22]. This capability gives rise to a new challenge, namely, the integration of a 3D vascular tree pattern model for bioprinting. Based on volumetric imaging of the natural prerequisite, vascular structures can be segmented and volume meshes can be generated [46] (Figure 5). However, most volumetric imaging techniques can only image vascular structures down to a certain size [46]. Smaller vessels cannot be detected using these imaging technologies [46]. Computed generation of detailed vascular trees is complex, considering the necessity to provide sufficient perfusion of all tissue regions. Mathematical approaches using best-fit algorithms for diffusion and consumption properties can be utilized to analyze the optimal size, distribution, and orientation of vascular structures [46,131]. Others developed a parametric, algorithm-based approach for the in silico design of bioartificial vascular trees [106,131]. Lee et al. solved the problem by application of an iterative approach [71]. First, magnetic resonance imaging was used to segment large blood vessels. Next, for a subset of the volume, the branching behavior of the large vessels was translated to the small volume and subsequently scaled down to engineer MRI-templated multiscale vasculature [71]. However, with further development of manufacturing technologies, blueprints for tissue-specific vascular trees might be necessary for computer-aided tissue engineering. This also highlights a different perspective on parenchymal tissue bioprinting, namely an end-to-end concept all the way from generative computer modeling to validation of biofabricated constructs in a standardized manner [106]. A future goal might be the characterization and fabrication of bioartificial vascular trees derived from parenchymal organ blueprints that show flow dynamics, mass transport, and, thus, ubiquitous oxygen and nutrient supply (Figure 5).

## 8. Discussion

Bioprinting may have the potential to overcome current challenges in bioartificial tissue engineering. One major limitation that studies still face is a lack of sufficient vascularization to supply oxygen and nutrients to effector cells [4]. State-of-the-art scaffold-based tissue engineering mostly relies on initial diffusional supply and is thus restricted in terms of the spatial dimension of the construct, the number of integrated effector cells, and long-term function. The evidence presented in this review suggests that fabrication of vascularized, functional parenchymal tissue might not be achieved using a single bioink and one bioprinting technology (Figure 4 and Figure 5). Direct printing of all elements including the capillary bed may not be feasible (Figure 4 and Figure 5). Vascular self-assembly strategies can be promoted by specific bioink formulations providing cues for vasculogenesis and angiogenesis. Such cues can be triggered by coculture with certain endothelial cell types and proangiogenic signaling molecules. Laser-assisted bioprinting one order of magnitude higher has the potential to fabricate delicate vascular structures. The combination of technologies enables generation of vascularized building blocks including effector cells [17,50]. The concept of sacrificial writing into functional tissue (SWIFT), based on previous developments of embedded bioprinting technologies such as FRESH, seems worthy of more extensive exploration. Beside SWIFT, bioprinting-assisted tissue emergence (BATE) utilizes organoid-building stem cells directly placing them in a bath of ECM materials and exploiting cellular self-assembly for tissue formation [132]. In perspective, bioprinting methods including materials mimicking the natural ECM and self-assembly of organoids producing the native ECM seem promising [132]. However, rather than depositing sacrificial ink in a bulk of vascularized building blocks with the intention of promoting bioactive lumen formation, direct coaxial extrusion of multilayered, hierarchical vessel-like structures might mimic nature more accurately down to a level of large arterioles and venules and might provide vascular connection anchors that can be anastomosed with the host circulation. A translation from experimental studies on the bench towards reproducible, streamlined processes for bedside application seams feasible, but remains to be developed. Strategies for vascularization of the construct need to be considered at all steps of the process before, during, and after printing (Figure 5). Post-printing cultivation can influence tissue and vessel maturation. Even though it is not within the scope of this review, extensive research showed that perfusion bioreactors allow dynamic cultivation of printed constructs, thus enabling mass transport through a viable matrix [133,134]. Pulsative flow in perfusion-based bioreactors encourages matrix formation and proliferation [135]. Furthermore, physical stimuli such as mechanical strain may positively influence tissue maturation [133,134].

**Figure 6:**
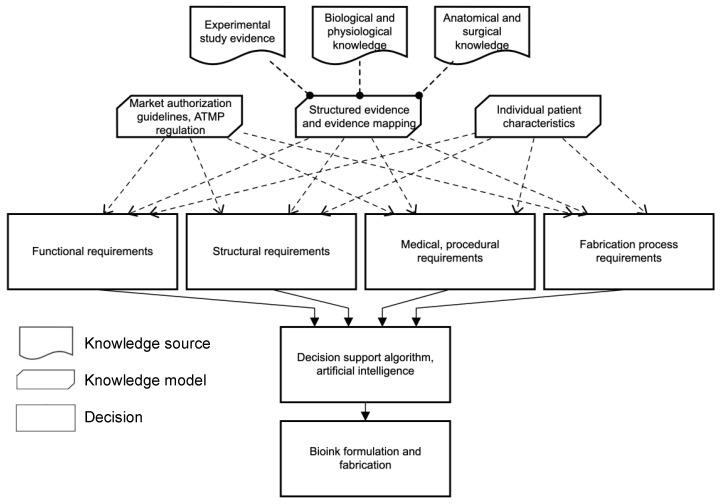
Decision model for bioink development. Evidence-driven or rather structured data-driven decision model and translation to an end-to-end process by decision model notation (DMN) for development of bioink for vascularization.

Bioprinting approaches for parenchymal organ engineering need to consider many parameters, each of which impedes standardization. Unlimited possibilities are offered by different bioprinting platforms with different technologies, by multiple, sometimes simultaneously utilized, bioink formulations of different materials and cells, and by the addition of signaling molecules. To date, structured evidence in the field of tissue engineering or bioprinting is not always easily accessible, thus hindering the identification of a suitable process for bioartificial organ engineering [7]. Many studies, therefore, rely on trial-and-error approaches investigating their respective objective. Instead, we propose standardized data-driven decision models for future research, e.g., regarding bioink development (Figure 6). The development of end-to-end processes from design to post-manufacturing validation are necessary for future ATMPs. We have identified the need for the structured reporting of research findings to enable sufficient data curation and structured analysis of existing evidence for further translation and application in bioink development (Figure 6). Computer simulation and in silico experiments can help to minimize trial-and-error approaches and define boundary conditions for models to be bioprinted [50]. Furthermore, there are already tendencies to address the challenges of multiparameter experimental concepts with the aid of algorithms, machine-learning approaches, and neural networks [136,137,138,139,140,141,142,143]. In the long term, artificial intelligence may support the design and manufacturing of bioartificial organs [136,137,138,139,140,141,142,143]. We envision data-driven artificial approaches to support all process stages of bioartificial tissue fabrication. Machine learning has been utilized as a powerful tool to decrypt the multitude of variables in existing study evidence, such as the exact formulations of bioink and the printing parameters for efficient experimental design. Based on natural, organ-specific vascular tree blueprints, algorithm-based approaches and validation by computer simulation can enable the creation of bioartificial vascular trees (Figure 5). Lastly, truly functional digital twins of tissues and organs can facilitate in silico bioprinting. This yields a perspective towards the customization of bioartificial tissue for patient-specific TEPs. The ultimate solution to the challenge of vascularization in bioprinting of parenchymal tissue requires further bioconvergence research.

## Data Availability

Not applicable.

## References

[1] Matai I., Kaur G., Seyedsalehi A., McClinton A., Laurencin C.T. (2020). Progress in 3D bioprinting technology for tissue/organ regenerative engineering. Biomaterials.

[2] Marchiori G., Berni M., Cassiolas G., Vivarelli L., Lopomo N.F., Fini M., Dallari D., Govoni M. (2021). Extra-Corporeal Membrane Oxygenation Cadaver Donors: What about Tissues Used as Allografts?. Membranes.

[3] Ortega-Deballon I., Hornby L., Shemie S.D. (2015). Protocols for uncontrolled donation after circulatory death: A systematic review of international guidelines, practices and transplant outcomes. Crit. Care.

[4] Salg G.A., Giese N.A., Schenk M., Huttner F.J., Felix K., Probst P., Diener M.K., Hackert T., Kenngott H.G. (2019). The emerging field of pancreatic tissue engineering: A systematic review and evidence map of scaffold materials and scaffolding techniques for insulin-secreting cells. J. Tissue Eng..

[5] Ramezankhani R., Torabi S., Minaei N., Madani H., Rezaeiani S., Hassani S.N., Gee A.P., Dominici M., Silva D.N., Baharvand H. (2020). Two Decades of Global Progress in Authorized Advanced Therapy Medicinal Products: An Emerging Revolution in Therapeutic Strategies. Front. Cell Dev. Biol..

[6] Oberweis C.V., Marchal J.A., Lopez-Ruiz E., Galvez-Martin P. (2020). A Worldwide Overview of Regulatory Frameworks for Tissue-Based Products. Tissue Eng. Part B Rev..

[7] Sekar M.P., Budharaju H., Zennifer A., Sethuraman S., Vermeulen N., Sundaramurthi D., Kalaskar D.M. (2021). Current standards and ethical landscape of engineered tissues-3D bioprinting perspective. J. Tissue Eng..

[8] Armoiry X., Cummins E., Connock M., Metcalfe A., Royle P., Johnston R., Rodrigues J., Waugh N., Mistry H. (2019). Autologous Chondrocyte Implantation with Chondrosphere for Treating Articular Cartilage Defects in the Knee: An Evidence Review Group Perspective of a NICE Single Technology Appraisal. Pharmacoeconomics.

[9] Carey J.L., Remmers A.E., Flanigan D.C. (2020). Use of MACI (Autologous Cultured Chondrocytes on Porcine Collagen Membrane) in the United States: Preliminary Experience. Orthop. J. Sports Med..

[10] Tilkin C., Duchesne B., Camby S. (2021). Holoclar(R), an autologous stem cells graft for sight recovery after ocular burns. Rev. Med. De Liege.

[11] Pellegrini G., Ardigo D., Milazzo G., Iotti G., Guatelli P., Pelosi D., De Luca M. (2018). Navigating Market Authorization: The Path Holoclar Took to Become the First Stem Cell Product Approved in the European Union. Stem Cells Transl. Med..

[12] Barbagli G., Akbarov I., Heidenreich A., Zugor V., Olianas R., Aragona M., Romano G., Balsmeyer U., Fahlenkamp D., Rebmann U. (2018). Anterior Urethroplasty Using a New Tissue Engineered Oral Mucosa Graft: Surgical Techniques and Outcomes. J. Urol..

[13] Murphy S.V., Atala A. (2014). 3D bioprinting of tissues and organs. Nat. Biotechnol..

[14] Schoneberg J., De Lorenzi F., Theek B., Blaeser A., Rommel D., Kuehne A.J.C., Kiessling F., Fischer H. (2018). Engineering biofunctional in vitro vessel models using a multilayer bioprinting technique. Sci. Rep..

[15] Sekine H., Okano T. (2020). Capillary Networks for Bio-Artificial Three-Dimensional Tissues Fabricated Using Cell Sheet Based Tissue Engineering. Int. J. Mol. Sci..

[16] Zhang Y., Yu Y., Akkouch A., Dababneh A., Dolati F., Ozbolat I.T. (2015). In Vitro Study of Directly Bioprinted Perfusable Vasculature Conduits. Biomater. Sci..

[17] De Moor L., Smet J., Plovyt M., Bekaert B., Vercruysse C., Asadian M., De Geyter N., Van Vlierberghe S., Dubruel P., Declercq H. (2021). Engineering microvasculature by 3D bioprinting of prevascularized spheroids in photo-crosslinkable gelatin. Biofabrication.

[18] McGuigan A.P., Sefton M.V. (2006). Vascularized organoid engineered by modular assembly enables blood perfusion. Proc. Natl. Acad. Sci. USA.

[19] Carmeliet P., Jain R.K. (2000). Angiogenesis in cancer and other diseases. Nature.

[20] Kang H.W., Lee S.J., Ko I.K., Kengla C., Yoo J.J., Atala A. (2016). A 3D bioprinting system to produce human-scale tissue constructs with structural integrity. Nat. Biotechnol..

[21] Barrs R.W., Jia J., Silver S.E., Yost M., Mei Y. (2020). Biomaterials for Bioprinting Microvasculature. Chem. Rev..

[22] Brumm P., Fritschen A., Doss L., Dorsam E., Blaeser A. (2022). Fabrication of biomimetic networks using viscous fingering in flexographic printing. Biomed. Mater..

[23] Accolla R.P., Simmons A.M., Stabler C.L. (2022). Integrating Additive Manufacturing (AM) Techniques to Improve Cell-Based Implants for the Treatment of Type 1 Diabetes. Adv. Healthc. Mater..

[24] Gungor-Ozkerim P.S., Inci I., Zhang Y.S., Khademhosseini A., Dokmeci M.R. (2018). Bioinks for 3D bioprinting: An overview. Biomater. Sci..

[25] Xu T., Kincaid H., Atala A., Yoo J.J. (2008). High-Throughput Production of Single-Cell Microparticles Using an Inkjet Printing Technology. J. Manuf. Sci. Eng..

[26] Blaeser A., Duarte Campos D.F., Puster U., Richtering W., Stevens M.M., Fischer H. (2016). Controlling Shear Stress in 3D Bioprinting is a Key Factor to Balance Printing Resolution and Stem Cell Integrity. Adv. Healthc. Mater..

[27] Lee V.K., Kim D.Y., Ngo H., Lee Y., Seo L., Yoo S.S., Vincent P.A., Dai G. (2014). Creating perfused functional vascular channels using 3D bio-printing technology. Biomaterials.

[28] Solis L.H., Ayala Y., Portillo S., Varela-Ramirez A., Aguilera R., Boland T. (2019). Thermal inkjet bioprinting triggers the activation of the VEGF pathway in human microvascular endothelial cells in vitro. Biofabrication.

[29] Lovecchio J., Cortesi M., Zani M., Govoni M., Dallari D., Giordano E. (2022). Fiber Thickness and Porosity Control in a Biopolymer Scaffold 3D Printed through a Converted Commercial FDM Device. Materials.

[30] Ioannidis K., Danalatos R.I., Champeris Tsaniras S., Kaplani K., Lokka G., Kanellou A., Papachristou D.J., Bokias G., Lygerou Z., Taraviras S. (2020). A Custom Ultra-Low-Cost 3D Bioprinter Supports Cell Growth and Differentiation. Front. Bioeng. Biotechnol..

[31] Chimene D., Kaunas R., Gaharwar A.K. (2020). Hydrogel Bioink Reinforcement for Additive Manufacturing: A Focused Review of Emerging Strategies. Adv. Mater..

[32] Ouyang L., Highley C.B., Sun W., Burdick J.A. (2017). A Generalizable Strategy for the 3D Bioprinting of Hydrogels from Nonviscous Photo-crosslinkable Inks. Adv. Mater..

[33] Koch L., Deiwick A., Chichkov B. (2021). Capillary-like Formations of Endothelial Cells in Defined Patterns Generated by Laser Bioprinting. Micromachines.

[34] Guillotin B., Souquet A., Catros S., Duocastella M., Pippenger B., Bellance S., Bareille R., Remy M., Bordenave L., Amedee J. (2010). Laser assisted bioprinting of engineered tissue with high cell density and microscale organization. Biomaterials.

[35] Guillemot F., Souquet A., Catros S., Guillotin B. (2010). Laser-assisted cell printing: Principle, physical parameters versus cell fate and perspectives in tissue engineering. Nanomedicine.

[36] Niklason L.E., Lawson J.H. (2020). Bioengineered human blood vessels. Science.

[37] Freeman S., Ramos R., Alexis Chando P., Zhou L., Reeser K., Jin S., Soman P., Ye K. (2019). A bioink blend for rotary 3D bioprinting tissue engineered small-diameter vascular constructs. Acta Biomater..

[38] Lawson J.H., Glickman M.H., Ilzecki M., Jakimowicz T., Jaroszynski A., Peden E.K., Pilgrim A.J., Prichard H.L., Guziewicz M., Przywara S. (2016). Bioengineered human acellular vessels for dialysis access in patients with end-stage renal disease: Two phase 2 single-arm trials. Lancet.

[39] Liu X., Wang X., Zhang L., Sun L., Wang H., Zhao H., Zhang Z., Liu W., Huang Y., Ji S. (2021). 3D Liver Tissue Model with Branched Vascular Networks by Multimaterial Bioprinting. Adv. Healthc. Mater..

[40] Kruger-Genge A., Blocki A., Franke R.P., Jung F. (2019). Vascular Endothelial Cell Biology: An Update. Int. J. Mol. Sci..

[41] Blaeser A., Duarte Campos D.F., Fischer H. (2017). 3D bioprinting of cell-laden hydrogels for advanced tissue engineering. Curr. Opin. Biomed. Eng..

[42] Fritschen A., Blaeser A. (2021). Biosynthetic, biomimetic, and self-assembled vascularized Organ-on-a-Chip systems. Biomaterials.

[43] Miller J.S., Stevens K.R., Yang M.T., Baker B.M., Nguyen D.H., Cohen D.M., Toro E., Chen A.A., Galie P.A., Yu X. (2012). Rapid casting of patterned vascular networks for perfusable engineered three-dimensional tissues. Nat. Mater..

[44] Dogan L., Scheuring R., Wagner N., Ueda Y., Schmidt S., Worsdorfer P., Groll J., Ergun S. (2021). Human iPSC-derived mesodermal progenitor cells preserve their vasculogenesis potential after extrusion and form hierarchically organized blood vessels. Biofabrication.

[45] Ribatti D., Vacca A., Nico B., Roncali L., Dammacco F. (2001). Postnatal vasculogenesis. Mech. Dev..

[46] Noor N., Shapira A., Edri R., Gal I., Wertheim L., Dvir T. (2019). 3D Printing of Personalized Thick and Perfusable Cardiac Patches and Hearts. Adv. Sci..

[47] Song K.H., Highley C.B., Rouff A., Burdick J.A. (2018). Complex 3D-Printed Microchannels within Cell-Degradable Hydrogels. Adv. Funct. Mater..

[48] Chiu Y.C., Cheng M.H., Engel H., Kao S.W., Larson J.C., Gupta S., Brey E.M. (2011). The role of pore size on vascularization and tissue remodeling in PEG hydrogels. Biomaterials.

[49] Bai F., Wang Z., Lu J., Liu J., Chen G., Lv R., Wang J., Lin K., Zhang J., Huang X. (2010). The correlation between the internal structure and vascularization of controllable porous bioceramic materials in vivo: A quantitative study. Tissue Eng. Part A.

[50] Salg G.A., Poisel E., Neulinger-Munoz M., Gerhardus J., Cebulla D., Bludszuweit-Philipp C., Vieira V., Nickel F., Herr I., Blaeser A. (2022). Toward 3D-bioprinting of an endocrine pancreas: A building-block concept for bioartificial insulin-secreting tissue. J. Tissue Eng..

[51] Han E.X., Wang J., Kural M., Jiang B., Leiby K.L., Chowdhury N., Tellides G., Kibbey R.G., Lawson J.H., Niklason L.E. (2021). Development of a Bioartificial Vascular Pancreas. J. Tissue Eng..

[52] Pedraza E., Coronel M.M., Fraker C.A., Ricordi C., Stabler C.L. (2012). Preventing hypoxia-induced cell death in beta cells and islets via hydrolytically activated, oxygen-generating biomaterials. Proc. Natl. Acad. Sci. USA.

[53] Moffat D., Ye K., Jin S. (2022). Decellularization for the retention of tissue niches. J. Tissue Eng..

[54] Pati F., Jang J., Ha D.H., Won Kim S., Rhie J.W., Shim J.H., Kim D.H., Cho D.W. (2014). Printing three-dimensional tissue analogues with decellularized extracellular matrix bioink. Nat. Commun..

[55] Lee J.H., Kim H.W. (2018). Emerging properties of hydrogels in tissue engineering. J. Tissue Eng..

[56] Yu C., Ma X., Zhu W., Wang P., Miller K.L., Stupin J., Koroleva-Maharajh A., Hairabedian A., Chen S. (2019). Scanningless and continuous 3D bioprinting of human tissues with decellularized extracellular matrix. Biomaterials.

[57] Soliman B.G., Major G.S., Atienza-Roca P., Murphy C.A., Longoni A., Alcala-Orozco C.R., Rnjak-Kovacina J., Gawlitta D., Woodfield T.B.F., Lim K.S. (2022). Development and Characterization of Gelatin-Norbornene Bioink to Understand the Interplay between Physical Architecture and Micro-Capillary Formation in Biofabricated Vascularized Constructs. Adv. Healthc. Mater..

[58] Wei S.Y., Chen T.H., Kao F.S., Hsu Y.J., Chen Y.C. (2022). Strategy for improving cell-mediated vascularized soft tissue formation in a hydrogen peroxide-triggered chemically-crosslinked hydrogel. J. Tissue Eng..

[59] Stratesteffen H., Kopf M., Kreimendahl F., Blaeser A., Jockenhoevel S., Fischer H. (2017). GelMA-collagen blends enable drop-on-demand 3D printablility and promote angiogenesis. Biofabrication.

[60] Muthusamy S., Kannan S., Lee M., Sanjairaj V., Lu W.F., Fuh J.Y.H., Sriram G., Cao T. (2021). 3D bioprinting and microscale organization of vascularized tissue constructs using collagen-based bioink. Biotechnol. Bioeng..

[61] Unagolla J.M., Jayasuriya A.C. (2020). Hydrogel-based 3D bioprinting: A comprehensive review on cell-laden hydrogels, bioink formulations, and future perspectives. Appl. Mater. Today.

[62] Moon J.J., Saik J.E., Poche R.A., Leslie-Barbick J.E., Lee S.H., Smith A.A., Dickinson M.E., West J.L. (2010). Biomimetic hydrogels with pro-angiogenic properties. Biomaterials.

[63] Son J., Hong S.J., Lim J.W., Jeong W., Jeong J.H., Kang H.W. (2021). Engineering Tissue-Specific, Multiscale Microvasculature with a Capillary Network for Prevascularized Tissue. Small Methods.

[64] Benning L., Gutzweiler L., Trondle K., Riba J., Zengerle R., Koltay P., Zimmermann S., Stark G.B., Finkenzeller G. (2018). Assessment of hydrogels for bioprinting of endothelial cells. J. Biomed. Mater. Res. A.

[65] Yamamura N., Sudo R., Ikeda M., Tanishita K. (2007). Effects of the mechanical properties of collagen gel on the in vitro formation of microvessel networks by endothelial cells. Tissue Eng..

[66] Mazzocchi A., Devarasetty M., Huntwork R., Soker S., Skardal A. (2018). Optimization of collagen type I-hyaluronan hybrid bioink for 3D bioprinted liver microenvironments. Biofabrication.

[67] Brandenberg N., Lutolf M.P. (2016). In Situ Patterning of Microfluidic Networks in 3D Cell-Laden Hydrogels. Adv. Mater..

[68] Gao G., Park J.Y., Kim B.S., Jang J., Cho D.W. (2018). Coaxial Cell Printing of Freestanding, Perfusable, and Functional In Vitro Vascular Models for Recapitulation of Native Vascular Endothelium Pathophysiology. Adv. Healthc. Mater..

[69] Farina M., Ballerini A., Fraga D.W., Nicolov E., Hogan M., Demarchi D., Scaglione F., Sabek O.M., Horner P., Thekkedath U. (2017). 3D Printed Vascularized Device for Subcutaneous Transplantation of Human Islets. Biotechnol. J..

[70] Weaver J.D., Headen D.M., Hunckler M.D., Coronel M.M., Stabler C.L., Garcia A.J. (2018). Design of a vascularized synthetic poly(ethylene glycol) macroencapsulation device for islet transplantation. Biomaterials.

[71] Lee A., Hudson A.R., Shiwarski D.J., Tashman J.W., Hinton T.J., Yerneni S., Bliley J.M., Campbell P.G., Feinberg A.W. (2019). 3D bioprinting of collagen to rebuild components of the human heart. Science.

[72] van Duinen V., Zhu D., Ramakers C., van Zonneveld A.J., Vulto P., Hankemeier T. (2019). Perfused 3D angiogenic sprouting in a high-throughput in vitro platform. Angiogenesis.

[73] Coronel M.M., Geusz R., Stabler C.L. (2017). Mitigating hypoxic stress on pancreatic islets via in situ oxygen generating biomaterial. Biomaterials.

[74] Coronel M.M., Liang J.P., Li Y., Stabler C.L. (2019). Oxygen generating biomaterial improves the function and efficacy of beta cells within a macroencapsulation device. Biomaterials.

[75] Liang J.P., Accolla R.P., Soundirarajan M., Emerson A., Coronel M.M., Stabler C.L. (2021). Engineering a macroporous oxygen-generating scaffold for enhancing islet cell transplantation within an extrahepatic site. Acta Biomater..

[76] Malda J., Visser J., Melchels F.P., Jungst T., Hennink W.E., Dhert W.J., Groll J., Hutmacher D.W. (2013). 25th anniversary article: Engineering hydrogels for biofabrication. Adv. Mater..

[77] Ahearne M. (2014). Introduction to cell-hydrogel mechanosensing. Interface Focus.

[78] Duarte Campos D.F., Zhang S., Kreimendahl F., Kopf M., Fischer H., Vogt M., Blaeser A., Apel C., Esteves-Oliveira M. (2020). Hand-held bioprinting for de novo vascular formation applicable to dental pulp regeneration. Connect. Tissue Res..

[79] Zhang T., Day J.H., Su X., Guadarrama A.G., Sandbo N.K., Esnault S., Denlinger L.C., Berthier E., Theberge A.B. (2019). Investigating Fibroblast-Induced Collagen Gel Contraction Using a Dynamic Microscale Platform. Front. Bioeng. Biotechnol..

[80] Chaudhuri O., Gu L., Klumpers D., Darnell M., Bencherif S.A., Weaver J.C., Huebsch N., Lee H.P., Lippens E., Duda G.N. (2016). Hydrogels with tunable stress relaxation regulate stem cell fate and activity. Nat. Mater..

[81] Wenz A., Tjoeng I., Schneider I., Kluger P.J., Borchers K. (2018). Improved vasculogenesis and bone matrix formation through coculture of endothelial cells and stem cells in tissue-specific methacryloyl gelatin-based hydrogels. Biotechnol. Bioeng..

[82] Harvey K., Welch Z., Kovala A.T., Garcia J.G., English D. (2002). Comparative analysis of in vitro angiogenic activities of endothelial cells of heterogeneous origin. Microvasc. Res..

[83] Skylar-Scott M.A., Uzel S.G.M., Nam L.L., Ahrens J.H., Truby R.L., Damaraju S., Lewis J.A. (2019). Biomanufacturing of organ-specific tissues with high cellular density and embedded vascular channels. Sci. Adv..

[84] Jia W., Gungor-Ozkerim P.S., Zhang Y.S., Yue K., Zhu K., Liu W., Pi Q., Byambaa B., Dokmeci M.R., Shin S.R. (2016). Direct 3D bioprinting of perfusable vascular constructs using a blend bioink. Biomaterials.

[85] Bosch-Rue E., Diez-Tercero L., Delgado L.M., Perez R.A. (2022). Biofabrication of Collagen Tissue-Engineered Blood Vessels with Direct Co-Axial Extrusion. Int. J. Mol. Sci..

[86] Bosch-Rue E., Delgado L.M., Gil F.J., Perez R.A. (2020). Direct extrusion of individually encapsulated endothelial and smooth muscle cells mimicking blood vessel structures and vascular native cell alignment. Biofabrication.

[87] Wang Y., Huang X., Shen Y., Hang R., Zhang X., Wang Y., Yao X., Tang B. (2019). Direct writing alginate bioink inside pre-polymers of hydrogels to create patterned vascular networks. J. Mater. Sci..

[88] Mirabella T., MacArthur J.W., Cheng D., Ozaki C.K., Woo Y.J., Yang M., Chen C.S. (2017). 3D-printed vascular networks direct therapeutic angiogenesis in ischaemia. Nat. Biomed. Eng..

[89] Wu P.K., Ringeisen B.R. (2010). Development of human umbilical vein endothelial cell (HUVEC) and human umbilical vein smooth muscle cell (HUVSMC) branch/stem structures on hydrogel layers via biological laser printing (BioLP). Biofabrication.

[90] Paek J., Park S.E., Lu Q., Park K.T., Cho M., Oh J.M., Kwon K.W., Yi Y.S., Song J.W., Edelstein H.I. (2019). Microphysiological Engineering of Self-Assembled and Perfusable Microvascular Beds for the Production of Vascularized Three-Dimensional Human Microtissues. ACS Nano.

[91] Olgasi C., Cucci A., Follenzi A. (2020). iPSC-Derived Liver Organoids: A Journey from Drug Screening, to Disease Modeling, Arriving to Regenerative Medicine. Int. J. Mol. Sci..

[92] Herland A., Maoz B.M., Das D., Somayaji M.R., Prantil-Baun R., Novak R., Cronce M., Huffstater T., Jeanty S.S.F., Ingram M. (2020). Quantitative prediction of human pharmacokinetic responses to drugs via fluidically coupled vascularized organ chips. Nat. Biomed. Eng..

[93] Lin N.Y.C., Homan K.A., Robinson S.S., Kolesky D.B., Duarte N., Moisan A., Lewis J.A. (2019). Renal reabsorption in 3D vascularized proximal tubule models. Proc. Natl. Acad. Sci. USA.

[94] Chuang C.H., Lin R.Z., Melero-Martin J.M., Chen Y.C. (2018). Comparison of covalently and physically cross-linked collagen hydrogels on mediating vascular network formation for engineering adipose tissue. Artif. Cells Nanomed. Biotechnol..

[95] Peters E.B., Christoforou N., Leong K.W., Truskey G.A., West J.L. (2016). Poly(ethylene glycol) Hydrogel Scaffolds Containing Cell-Adhesive and Protease-Sensitive Peptides Support Microvessel Formation by Endothelial Progenitor Cells. Cell. Mol. Bioeng..

[96] Szepes M., Melchert A., Dahlmann J., Hegermann J., Werlein C., Jonigk D., Haverich A., Martin U., Olmer R., Gruh I. (2020). Dual Function of iPSC-Derived Pericyte-Like Cells in Vascularization and Fibrosis-Related Cardiac Tissue Remodeling In Vitro. Int. J. Mol. Sci..

[97] Sances S., Ho R., Vatine G., West D., Laperle A., Meyer A., Godoy M., Kay P.S., Mandefro B., Hatata S. (2018). Human iPSC-Derived Endothelial Cells and Microengineered Organ-Chip Enhance Neuronal Development. Stem Cell Rep..

[98] Rajendran P., Rengarajan T., Thangavel J., Nishigaki Y., Sakthisekaran D., Sethi G., Nishigaki I. (2013). The vascular endothelium and human diseases. Int. J. Biol. Sci..

[99] Kosyakova N., Kao D.D., Figetakis M., Lopez-Giraldez F., Spindler S., Graham M., James K.J., Won Shin J., Liu X., Tietjen G.T. (2020). Differential functional roles of fibroblasts and pericytes in the formation of tissue-engineered microvascular networks in vitro. NPJ Regen. Med..

[100] Caplan A.I. (2017). Mesenchymal Stem Cells: Time to Change the Name!. Stem Cells Transl. Med..

[101] Mayer H., Bertram H., Lindenmaier W., Korff T., Weber H., Weich H. (2005). Vascular endothelial growth factor (VEGF-A) expression in human mesenchymal stem cells: Autocrine and paracrine role on osteoblastic and endothelial differentiation. J. Cell Biochem..

[102] Ortega I., Dew L., Kelly A.G., Chong C.K., MacNeil S., Claeyssens F. (2015). Fabrication of biodegradable synthetic perfusable vascular networks via a combination of electrospinning and robocasting. Biomater. Sci..

[103] Contessi Negrini N., Bonnetier M., Giatsidis G., Orgill D.P., Fare S., Marelli B. (2019). Tissue-mimicking gelatin scaffolds by alginate sacrificial templates for adipose tissue engineering. Acta Biomater..

[104] Homan K.A., Kolesky D.B., Skylar-Scott M.A., Herrmann J., Obuobi H., Moisan A., Lewis J.A. (2016). Bioprinting of 3D Convoluted Renal Proximal Tubules on Perfusable Chips. Sci. Rep..

[105] Kolesky D.B., Homan K.A., Skylar-Scott M.A., Lewis J.A. (2016). Three-dimensional bioprinting of thick vascularized tissues. Proc. Natl. Acad. Sci. USA.

[106] Forget A., Derme T., Mitterberger D., Heiny M., Sweeney C., Mudili L., Dargaville T.R., Shastri V.P. (2019). Architecture-inspired paradigm for 3D bioprinting of vessel-like structures using extrudable carboxylated agarose hydrogels. Emergent Mater..

[107] Wu W., DeConinck A., Lewis J.A. (2011). Omnidirectional printing of 3D microvascular networks. Adv. Mater..

[108] Bertassoni L.E., Cecconi M., Manoharan V., Nikkhah M., Hjortnaes J., Cristino A.L., Barabaschi G., Demarchi D., Dokmeci M.R., Yang Y. (2014). Hydrogel bioprinted microchannel networks for vascularization of tissue engineering constructs. Lab Chip.

[109] Li S., Liu Y.Y., Liu L.J., Hu Q.X. (2016). A Versatile Method for Fabricating Tissue Engineering Scaffolds with a Three-Dimensional Channel for Prevasculature Networks. ACS Appl. Mater. Interfaces.

[110] Davoodi E., Montazerian H., Zhianmanesh M., Abbasgholizadeh R., Haghniaz R., Baidya A., Pourmohammadali H., Annabi N., Weiss P.S., Toyserkani E. (2022). Template-Enabled Biofabrication of Thick 3D Tissues with Patterned Perfusable Macrochannels. Adv. Healthc. Mater..

[111] Millik S.C., Dostie A.M., Karis D.G., Smith P.T., McKenna M., Chan N., Curtis C.D., Nance E., Theberge A.B., Nelson A. (2019). 3D printed coaxial nozzles for the extrusion of hydrogel tubes toward modeling vascular endothelium. Biofabrication.

[112] Fleischer S., Tavakol D.N., Vunjak-Novakovic G. (2020). From arteries to capillaries: Approaches to engineering human vasculature. Adv. Funct. Mater..

[113] Heintz K.A., Bregenzer M.E., Mantle J.L., Lee K.H., West J.L., Slater J.H. (2016). Fabrication of 3D Biomimetic Microfluidic Networks in Hydrogels. Adv. Healthc. Mater..

[114] Hinton T.J., Jallerat Q., Palchesko R.N., Park J.H., Grodzicki M.S., Shue H.J., Ramadan M.H., Hudson A.R., Feinberg A.W. (2015). Three-dimensional printing of complex biological structures by freeform reversible embedding of suspended hydrogels. Sci. Adv..

[115] Shiwarski D.J., Hudson A.R., Tashman J.W., Feinberg A.W. (2021). Emergence of FRESH 3D printing as a platform for advanced tissue biofabrication. APL Bioeng..

[116] Blaeser A., Duarte Campos D.F., Weber M., Neuss S., Theek B., Fischer H., Jahnen-Dechent W. (2013). Biofabrication under fluorocarbon: A novel freeform fabrication technique to generate high aspect ratio tissue-engineered constructs. BioResearch Open Access.

[117] Highley C.B., Rodell C.B., Burdick J.A. (2015). Direct 3D Printing of Shear-Thinning Hydrogels into Self-Healing Hydrogels. Adv. Mater..

[118] Paszkowiak J.J., Dardik A. (2003). Arterial wall shear stress: Observations from the bench to the bedside. Vasc. Endovasc. Surg..

[119] Liu X., Carter S.D., Renes M.J., Kim J., Rojas-Canales D.M., Penko D., Angus C., Beirne S., Drogemuller C.J., Yue Z. (2019). Development of a Coaxial 3D Printing Platform for Biofabrication of Implantable Islet-Containing Constructs. Adv. Healthc. Mater..

[120] Zhang Y., Yu Y., Chen H., Ozbolat I.T. (2013). Characterization of printable cellular micro-fluidic channels for tissue engineering. Biofabrication.

[121] Pi Q., Maharjan S., Yan X., Liu X., Singh B., van Genderen A.M., Robledo-Padilla F., Parra-Saldivar R., Hu N., Jia W. (2018). Digitally Tunable Microfluidic Bioprinting of Multilayered Cannular Tissues. Adv. Mater..

[122] Skylar-Scott M.A., Mueller J., Visser C.W., Lewis J.A. (2019). Voxelated soft matter via multimaterial multinozzle 3D printing. Nature.

[123] Liu W., Zhang Y.S., Heinrich M.A., De Ferrari F., Jang H.L., Bakht S.M., Alvarez M.M., Yang J., Li Y.C., Trujillo-de Santiago G. (2017). Rapid Continuous Multimaterial Extrusion Bioprinting. Adv. Mater..

[124] Mirdamadi E., Muselimyan N., Koti P., Asfour H., Sarvazyan N. (2019). Agarose Slurry as a Support Medium for Bioprinting and Culturing Freestanding Cell-Laden Hydrogel Constructs. 3d Print. Addit. Manuf..

[125] Farina M., Chua C.Y.X., Ballerini A., Thekkedath U., Alexander J.F., Rhudy J.R., Torchio G., Fraga D., Pathak R.R., Villanueva M. (2018). Transcutaneously refillable, 3D-printed biopolymeric encapsulation system for the transplantation of endocrine cells. Biomaterials.

[126] Smink A.M., Li S., Hertsig D.T., de Haan B.J., Schwab L., van Apeldoorn A.A., de Koning E., Faas M.M., Lakey J.R., de Vos P. (2017). The Efficacy of a Prevascularized, Retrievable Poly(D,L,-lactide-co-epsilon-caprolactone) Subcutaneous Scaffold as Transplantation Site for Pancreatic Islets. Transplantation.

[127] Laschke M.W., Rucker M., Jensen G., Carvalho C., Mulhaupt R., Gellrich N.C., Menger M.D. (2008). Improvement of vascularization of PLGA scaffolds by inosculation of in situ-preformed functional blood vessels with the host microvasculature. Ann. Surg..

[128] Wiseman S.M., Memarnejadian A., Boyce G.K., Nguyen A., Walker B.A., Holmes D.T., Welch I.D., Mazzuca D.M., Toleikis P.M. (2022). Subcutaneous transplantation of human thyroid tissue into a pre-vascularized Cell Pouch device in a Mus musculus model: Evidence of viability and function for thyroid transplantation. PLoS ONE.

[129] Pepper A.R., Pawlick R., Gala-Lopez B., MacGillivary A., Mazzuca D.M., White D.J., Toleikis P.M., Shapiro A.M. (2015). Diabetes Is Reversed in a Murine Model by Marginal Mass Syngeneic Islet Transplantation Using a Subcutaneous Cell Pouch Device. Transplantation.

[130] Pepper A.R., Pawlick R., Bruni A., Wink J., Rafiei Y., O’Gorman D., Yan-Do R., Gala-Lopez B., Kin T., MacDonald P.E. (2017). Transplantation of Human Pancreatic Endoderm Cells Reverses Diabetes Post Transplantation in a Prevascularized Subcutaneous Site. Stem Cell Rep..

[131] Jessen E., Steinbach M.C., Debbaut C., Schillinger D. (2022). Rigorous mathematical optimization of synthetic hepatic vascular trees. J. R Soc. Interface.

[132] Brassard J.A., Nikolaev M., Hubscher T., Hofer M., Lutolf M.P. (2021). Recapitulating macro-scale tissue self-organization through organoid bioprinting. Nat. Mater..

[133] Zhang Y.S., Arneri A., Bersini S., Shin S.R., Zhu K., Goli-Malekabadi Z., Aleman J., Colosi C., Busignani F., Dell’Erba V. (2016). Bioprinting 3D microfibrous scaffolds for engineering endothelialized myocardium and heart-on-a-chip. Biomaterials.

[134] Meinert C., Schrobback K., Hutmacher D.W., Klein T.J. (2017). A novel bioreactor system for biaxial mechanical loading enhances the properties of tissue-engineered human cartilage. Sci. Rep..

[135] Govoni M., Muscari C., Guarnieri C., Giordano E. (2013). Mechanostimulation protocols for cardiac tissue engineering. Biomed. Res. Int..

[136] Abdollahi S., Davis A., Miller J.H., Feinberg A.W. (2018). Expert-guided optimization for 3D printing of soft and liquid materials. PLoS ONE.

[137] Malekpour A., Chen X. (2022). Printability and Cell Viability in Extrusion-Based Bioprinting from Experimental, Computational, and Machine Learning Views. J. Funct. Biomater..

[138] Shin J., Lee Y., Li Z., Hu J., Park S.S., Kim K. (2022). Optimized 3D Bioprinting Technology Based on Machine Learning: A Review of Recent Trends and Advances. Micromachines.

[139] Fu Z., Angeline V., Sun W. (2021). Evaluation of Printing Parameters on 3D Extrusion Printing of Pluronic Hydrogels and Machine Learning Guided Parameter Recommendation. Int. J. Bioprinting.

[140] Tian S., Stevens R., McInnes B.T., Lewinski N.A. (2021). Machine Assisted Experimentation of Extrusion-Based Bioprinting Systems. Micromachines.

[141] An J., Chua C.K., Mironov V. (2021). Application of Machine Learning in 3D Bioprinting: Focus on Development of Big Data and Digital Twin. Int. J. Bioprinting.

[142] Bone J.M., Childs C.M., Menon A., Poczos B., Feinberg A.W., LeDuc P.R., Washburn N.R. (2020). Hierarchical Machine Learning for High-Fidelity 3D Printed Biopolymers. ACS Biomater. Sci. Eng..

[143] Kim J., McKee J.A., Fontenot J.J., Jung J.P. (2019). Engineering Tissue Fabrication With Machine Intelligence: Generating a Blueprint for Regeneration. Front. Bioeng. Biotechnol..

